# Psychoactive Effects of *Lactobacillus johnsonii* BS15 on Preventing Memory Dysfunction Induced by Acute Ethanol Exposure Through Modulating Intestinal Microenvironment and Improving Alcohol Metabolic Level

**DOI:** 10.3389/fmicb.2022.847468

**Published:** 2022-04-01

**Authors:** Ning Sun, Bin Zhu, Jinge Xin, Lianxin Li, Baoxing Gan, Xi Cao, Jing Fang, Kangcheng Pan, Bo Jing, Yan Zeng, Cheng Lv, Ling Zhao, Dong Zeng, Peng Xu, Hesong Wang, Xueqin Ni

**Affiliations:** ^1^Animal Microecology Institute, College of Veterinary Medicine, Sichuan Agricultural University, Chengdu, China; ^2^MOE Key Laboratory for Neuroinformation, Center for Information in Medicine, The School of Life Sciences and Technology, Clinical Hospital of Chengdu Brain Science Institute, University of Electronic Science and Technology of China, Chengdu, China; ^3^Guangzhou Beneco Biotechnology Co., Ltd., Guangzhou, China; ^4^Key Laboratory of Animal Diseases and Environmental Hazards of Sichuan Province, College of Veterinary Medicine, Sichuan Agricultural University, Chengdu, China; ^5^Department of Pharmacy, College of Veterinary Medicine, Sichuan Agricultural University, Chengdu, China

**Keywords:** abuse ethanol, *Lactobacillus johnsonii* BS15, memory impairment, microbiota-gut-brain axis, hippocampus

## Abstract

The negative effects of ethanol (EtOH) abuse on the body have been widely reported in recent years. Building on the microbiota-gut-brain axis hypothesis, our study aimed to demonstrate the potential psychobiotic role of *Lactobacillus johnsonii* BS15 in the preventive effects of acute EtOH intake on memory impairment. We also determined whether *L. johnsonii* BS15 intake could effectively improve resistance to acute drinking and alleviate the adverse effects of EtOH. Male mice were fed *L. johnsonii* BS15 orally with (Probiotic group) or without (Control and Alcohol groups) daily dose of 0.2 × 10^9^ CFU/ml per mouse for 28 days. Gavage with *L. johnsonii* BS15 significantly modified the ileal microbial ecosystem (assessed by 16S rRNA gene sequencing) in favor of Firmicutes and *Lactobacillus*, indicating the ability of BS15 to restore the gut microbiota. The acute EtOH exposure model (7 g/kg EtOH per mice) was established by gavage, which was administered to the alcohol and probiotic groups on day 28 of the experiment. The *L. johnsonii* BS15 intake effectively reduced alcohol unconsciousness time, blood alcohol concentration, and serum aspartate aminotransferase (AST) and alanine aminotransferase (ALT) levels. Meanwhile, the improvement of ethanol resistance time and the activities of alcohol dehydrogenase (ADH) and aldehyde dehydrogenase (ALDH) in the liver were shown by BS15 in acute alcohol-induced mice. We found that acute EtOH exposure reduced the exploration ratio (assessed by the novel object recognition test), escape latency, number of errors (assessed by passive avoidance test), and spontaneous exploration (assessed by T-maze test) in mice, which were obviously improved by *L. johnsonii* BS15. In the hippocampus, *L. johnsonii* BS15 significantly reversed the decrease in antioxidant capacity of superoxide dismutase (SOD), malondialdehyde (MDA), and glutathione (GSH) and mRNA expression of memory-related functional proteins of brain-derived neurotrophic factor (BDNF) and cyclic ampresponse element binding protein (CREB) in the hippocampal tissue after acute EtOH exposure. In conclusion, *L. johnsonii* BS15 intake appears as a promising psychoactive therapy to ameliorate alcohol-mediated memory impairment by increasing EtOH metabolic levels.

## Introduction

Alcoholism is an alarming global issue (Neyrinck et al., 2017). Alcohol-related injuries are one of the most common preventable diseases worldwide, with 3.3 million deaths, accounting for 6% of all global deaths (World Health Organization, 2014; Asrani et al., 2019). It is well known that alcohol abuse can damage multiple organs, mainly the liver, intestine, and brain (Bajaj, 2019). Studies have shown that long-term drinking can change the intestinal microbial composition, damage the intestinal mucosal barrier, and destroy intestinal homeostasis (Stärkel and Schnabl, 2016). Additionally, dopamine, glutamate, and γ-changes in aminobutyric acid (GABA) release are associated with excessive alcohol exposure (Alasmari et al., 2018). These neuroadaptations of psychoneuroimmunity promote mood regulation disorders and further trigger inflammation, leading to alcohol use disorder (AUD) and related affective comorbidities (Koob, 2013; Hillemacher et al., 2018). Based on preclinical studies, there is increasing evidence that alcoholism negatively affects brain memory and cognitive function (Scholey et al., 2019). A recent study reported that alcohol addiction could promote rapid acetylation of histones in the brain, thereby affecting the expression of learning and memory genes (Mews et al., 2019). Moreover, long-term intake of high-dose alcohol can induce cognitive impairment, improve the activity of β-amyloid precursor protein (β-APP)-related enzymes, increase the content and deposition of amyloid β-protein (Aβ), and trigger the progression of Alzheimer’s disease (AD) (Gong et al., 2021). Unfortunately, the exact mechanism of alcohol-induced memory and cognitive impairment remains unclear, making it difficult to prevent acute or chronic ethanol exposure.

The microbiota, comprising trillions of microbes in the gut, has become a major driver of the gut-brain axis (GBA). Most surprisingly, the microbiota largely shapes the structure and function of the nervous system (Cryan et al., 2019). Perhaps the most specific effect of the microbiota on the host is the regulation of brain physiology and behavior (Hao et al., 2019). Over the past two decades, explosive research has been conducted in the field of microbiota-host interaction. Symbiotic microorganisms have been shown to alter host neurophysiology and pathophysiology, leading to changes in neurological and mental diseases, including depression, anxiety, and social behavior (Sharon et al., 2016; Vuong et al., 2017; Cryan et al., 2019; Fang et al., 2020; Morais et al., 2021). Interestingly, probiotics, composed of different strains of lactic acid bacteria and/or *Bifidobacteria*, have been shown to affect the behavior of mice, including reduced anxiety-related symptoms (Bravo et al., 2011; Messaoudi et al., 2011; Mohammadi et al., 2016) and improved memory (Smith et al., 2014; O’Hagan et al., 2017). The use of probiotic strains can improve the memory of objects and object positions (Smith et al., 2014; Liang et al., 2015; Warburton and Brown, 2015; O’Hagan et al., 2017), rather than object chronological memory (O’Hagan et al., 2017). By analyzing the metabolic characteristics of specific lactic acid bacteria strains in the mouse brain, metabolic clues to enhance memory have been identified. However, metabolic mediators, if any, between the gut and brain remain unknown. To date, it is certain that reduction of the intestinal microbial community can prevent alcohol-induced neuroinflammation, resulting in changes in the expression of inflammatory bodies in the intestine and brain (Lowe et al., 2018). In conclusion, the interaction between alcohol-related brain dysfunction and communication *via* the gut microbiota axis remains unclear. Therefore, more animal experimental evidence is needed to prove the relationship between intestinal flora and the hippocampus under different conditions.

*Lactobacillus johnsonii* BS15 (CCTCC M2013663) was isolated from self-made yogurt in Hongyuan Prairie, Aba Autonomous Prefecture, China (Xin et al., 2014). The potential probiotic effects of *L. johnsonii* BS15 have been demonstrated in our previous studies (Xin et al., 2014; Sun et al., 2020). A recent study (Wang et al., 2021) reported that BS15 could resist demyelination and neuroinflammation in the brain by reducing impairment and improving fluoride-induced memory dysfunction. Therefore, we studied the effects of acute ethanol (EtOH) exposure and supplementation with *L. johnsonii* BS15 on hippocampal memory and cognitive functions in mice using high-throughput sequencing, behavioral testing, and biochemical analysis. From the perspective of the gut-brain axis, it is unclear whether *L. johnsonii* BS15 reconstructs the community composition and diversity in the intestinal microbiome before alcohol abuse and whether different gut species patterns could effectively prevent liver injury and memory dysfunction in mice after acute EtOH exposure. Therefore, under the same experimental conditions, combining these two aspects in animal experiments could explore the influence of excessive EtOH intake and beneficial treatment on alcohol metabolism and hippocampal memory function.

## Materials and Methods

### Culture and Treatment With BS15

In this study, *L. johnsonii* BS15 was cultured anaerobically in de Man, Rogosa, and Sharpe (MRS) broth (QDRS Biotec, Qingdao, Shandong, China). The plate count method was used to count the bacterial cells. Briefly, the bacterial solution was diluted 10 times with phosphate-buffered saline (PBS) (pH 7.0). Dilution gradients of 10^–5^, 10^–6^, and 10^–7^ were chosen, and 10 μl of each gradient was drawn to drop onto the MRS agar medium and repeated thrice. MRS agar medium was then cultured for 36 h at 37°C. An appropriate gradient (easy to count bacteria) was selected for bacterial counting. Next, centrifugation (3,000 rpm, 4°C, 15 min) and washing were done to separate the cells from the cultures and were resuspended in PBS for experimental use. The concentration of the suspension was 1 × 10^9^ CFU BS15/ml (daily consumption dose: 0.2 ml/mice; Xin et al., 2014).

### Study Design and Animal Treatment

**FIGURE 1:**
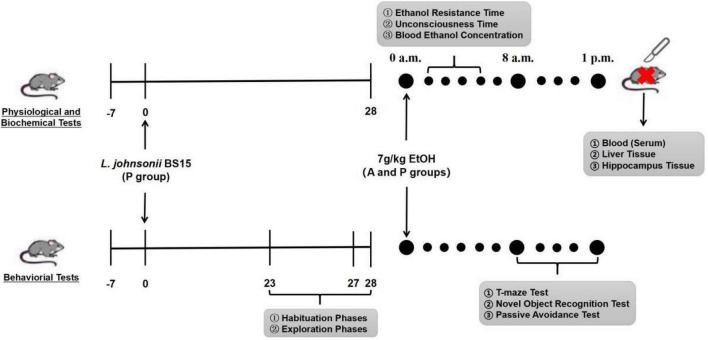
Flow diagram of experimental design. Mice were infected with EtOH (7 g/kg mice) at 28th day after orogastric challenge with either *L. johnsonii* BS15 suspension or PBS solution. Habituation, exploration, and training phase of behavioral tasks were conducted at 23rd day (T-maze test), 27th day (passive avoidance test), and 28th day (novel object recognition test), respectively, then the testing phase of all behavioral tasks was performed about 8 h after acute EtOH exposure at 28th day; 8–10 mice in each group were required to participate in T-maze test, novel object recognition test, and passive avoidance test, respectively. Besides, blood ethanol concentration, ethanol resistance, and unconsciousness time were evaluated by additional 6–10 mice in each group in the presence of acute EtOH exposure. In the end, blood, liver, and hippocampal tissues were collected to measure biochemical tests.

A total of 146 male Institute of Cancer Research (ICR) mice (22 ± 2 g) were purchased from the Chengdu Dashuo Biological Research Institute (Chengdu, Sichuan, China). All animals were fed normally for 7 days (adaptation period). In this study, the first day after the adaptation period was defined as day 1, as the experiment started. Mice were divided into three groups: control (C), alcohol (A), and probiotics (P). The mice in the P group were fed (gavage) the suspension of *L. johnsonii* BS15 once a day at 8 a.m (day 1–day 28). Groups C and A were treated with an equal PBS volume. All animals were kept in a room where temperature (22 ± 2°C) and a 12-h/12-h light/dark cycle (dark period: 7 p.m.–7 a.m.) could be controlled. The acute EtOH exposure model was induced by a single intragastric administration of 32% (v/v) EtOH at a dose of 7 g/kg on day 28 in the A and P groups (Carson and Pruett, 1996). Group C was treated with the same PBS volume. All animal experiments were performed in accordance with the Guidelines for the Care and Use of Laboratory Animals and approved by the Institutional Animal Care and Use Committee of Sichuan Agricultural University (approval number: SYXKchuan2019–187). All behavioral tasks were conducted between 7 a.m. and 1 p.m. Figure 1 illustrates the experimental design of this study.

### Measurement of Ethanol Resistance Time, Unconsciousness Time, and Blood Ethanol Concentration

On day 28 of the experiment, 6–10 mice from A and P groups were selected for the measurement of ethanol resistance time, unconsciousness time, and blood ethanol concentration after acute EtOH exposure.

Ethanol resistance time was measured as the time interval between acute exposure to EtOH and the loss of righting reflex. Unconsciousness time was measured as the duration of loss of righting reflex. In our study, mice were administered a high dose of EtOH and were determined to have lost the righting reflex when ataxia developed. Meanwhile, the loss of return to normal reflex time was evaluated as the time taken for the animals to return to normal. At 30, 60, 90, and 150 min after acute EtOH exposure, the blood of the orbital venous plexus was taken from a heparinized tube. Subsequently, the blood samples were centrifuged at 1,000 × *g* at 4°C for 15 min (Chen et al., 2014).

### Passive Avoidance Test

#### Habituation and Training Phase

On day 27 of the experiment, 10 mice from each group were put into the central platform to adapt to the testing device, and then, the grid was powered on (36 V, 50 Hz, 1 mA, 2 s) for 3 min to train. When the mouse’s foot touches the grid, the animal can obtain an electric shock that keeps it on the central platform. The mice were then put back into the cage, and the testing phase was conducted 24 h later.

During the testing phase, the mice, approximately 8 h after acute EtOH exposure, were placed on the central platform of the testing device again and were required to stay for 3 min. Subsequently, the grid in the device was powered during the testing period. Briefly, the interval when the animal walked off the platform for the first time was denoted as escape latency. In addition, the total number of times the mice left the platform within 3 min was recorded as the time of error. Therefore, shorter escape latency or greater error times in mice indicate memory dysfunction (Chen et al., 2014).

### T-Maze

On day 23 of the experiment, eight mice from each group were selected for the T-maze test. We conducted a T-maze test on the animals based on the method outlined by Deacon and Rawlins (2006). This included a feeding point for food provided at the distal end of each goal arm. The proximal end had a regulable door for controlling the open or closed goal arm. Besides, the food made up of 1:1 (v/v) water/full-fat sweetened condensed milk (Nestle, Qingdao, Shandong, China) was provided as a reward, of which 0.07 ml was given per trial by preset pipette.

#### Habituation and Exploration Phases

During the habituation phase (2 days), animals were softly stroked five times every day (3 min per time) to make the mice adapt to the smell and touch from the experimenters. The mice were then fed with reward food (0.5 ml) each day to reduce their fear of new things. Finally, the exploration phase (3 days) was conducted after the habituation phase, and mice were placed into a testing apparatus in which two goal arms were opened and allowed to move freely for 10 min. Subsequently, the mice were allowed to explore from the start arm to one goal arm (no more than 3 min), where the reward food was given to the food point. Simultaneously, the other goal arm was closed by the door. Each mouse was trained four times every day (equal numbers of left and right goal arms opened).

#### Testing Phase

One goal arm of the apparatus was closed, while the other goal arm remained open (equal times of left and right goal arm) and provided rewarding food at the corresponding point. One mouse, approximately 8 h after acute EtOH exposure, was allowed to explore freely in the apparatus for no more than 3 min and was subsequently removed. After waiting for 0 or 1 min, the mouse was again placed in the maze device. At the same time, the previously closed goal arm was opened, and rewarding food was given at the food point. If different goal arms were entered by the same mice in the two trials, it was recorded as correct. If the same goal arm was entered twice continuously, it was recorded as an error. Every mouse underwent the same process for 10 rounds (Deacon and Rawlins, 2006; Gareau et al., 2011).

### Novel Object Recognition Test

The test procedure consisted of three phases: habituation, exploration, and testing (Antunes and Biala, 2012). After 28 days of experimentation, 10 mice from each group were selected for the novel object recognition test (NOR).

#### Habituation and Exploration Phase

Each mouse was allowed to move freely in the open-field area (l × b × h = 40 × 40 × 45 cm) for 1 h in the absence of objects. The mouse was then removed from the area and placed in its home cage. Each mouse was placed in the area to freely explore two different objects (#A and #B) for 5 min during the familiarization phase. The two objects were placed at opposite positions in the cage.

#### Testing Phase

The mouse was given an intermediate retention interval of 20 min and then placed in the same area and re-exposed to object (#B) along with a new object (#C, distinguishable from object #A). The exploration ratio [F#C/(F#C + F#B) × 100, where F#C = frequency of exploring object #C, and F#B = frequency of exploring object #B] was calculated to evaluate memory function. The objects used in this study included a gray hard stone (#A), white hard stone (#B), and small blue cap (#C).

### Biochemical Analysis

On day 28 of this study, six mice from each treatment group were selected for biochemical measurements approximately 8 h after acute EtOH exposure. The hippocampus, liver, and blood were collected, and the serum was separated. The liver and part of the hippocampus were collected. Serum and tissue supernatants were prepared for subsequent biochemical analysis. The activities of aspartate aminotransferase (AST) and alanine aminotransferase (ALT) in the serum and the activities of superoxide dismutase (SOD), glutathione peroxidase (GSH-Px), GSH, and malondialdehyde (MDA) in the hippocampus were determined using kits purchased from Nanjing Jiancheng Institute of Biological Engineering (Nanjing, China). ADH and ALDH levels in the liver were determined using kits purchased from Abcam (Cambridge, MA, United States). The detailed procedures followed the manufacturer’s instructions, using different reagent kits.

### Real-Time Quantitative Polymerase Chain Reaction Analysis of Gene Expression

Residual hippocampal RNA during the above sampling process was extracted using E.Z.N.A.^®^ Total RNA Kit (OMEGA Bio-Tek, Doraville, GA, United States) based on the method outlined by product instructions. The total RNA was detected by the ratio of absorbance at 260 nm and 280 nm and by agarose gel electrophoresis for quantitative and qualitative assessments. Then, the total RNA was transcribed into first-strand complementary DNA (cDNA) using the PrimeScript RT reagent kit with gDNA Eraser (Thermo Scientific, Waltham, MA, United States) according to the method outlined by the manufacturer’s instructions. Finally, the cDNA products were stored at -80°C until further study. Real-time quantitative polymerase chain reaction (RT-qPCR) test was conducted using LightCycle 96 Real-Time System (Boehringer Mannheim GmbH, Germany) and SYBR Green Supermix (Bio-Rad, Hercules, CA, United States) to detect the relative expression levels of memory-related functional proteins in the hippocampus tissue. The reaction mixture (10 μl) included cDNA products (1 μl), forward and reverse primers (2 μl), SYBR Green Supermix (5 μl), and sterile deionized water (2 μl). The thermocycling protocol was as follows: 5 min at 95°C, followed by 40 cycles of 10 s denaturation at 95°C, and 30 s annealing/extension at the optimum temperature (Table 1). A melting curve analysis was performed to monitor the purity of the PCR product. β-actin was used to normalize the relative mRNA expression levels of target genes, with values presented as 2^–ΔΔCq^. Primer sequences and optimum annealing temperatures are listed in Table 1 (Niu et al., 2018).

**TABLE 1:** Primer sequences for RT-qPCR in hippocampus.

Gene	Tm (^°^C)	Sequence
β-actin	60	Forward: GCTCTTTTCCAGCCTTCCTT Reverse: GATGTCAACGTCACACTT
BDNF	60	Forward: GCGCCCATGAAAGAAGTAAA Reverse: TCGTCAGACCTCTCGAACCT
CREB	60	Forward: CCAGTTGCAAACATCAGTGG Reverse: TTGTGGGCATGAAGCAGTAG
NCAM	60	Forward: GGGAACTCCATCAAGGTGAA Reverse: TTGAGCATGACGTGGACACT
c-Fos	59.5	Forward: CAGAGCGGGAATGGTGAAGA Reverse: CTGTCTCCGCTTGGAGTGTA

### Intestinal Microbial Compositions in the Ileum

Before establishing the acute EtOH exposure model, the ileal contents of six mice were collected from the C and P groups for 16S rRNA gene sequencing.

Microbial DNA was obtained using an E.Z.N.A.TM stool DNA isolation kit (Omega Bio-Tek, Doraville, CA, United States). The final elution volume was 100 μl, and the purity, concentration, integrity, and fragment size were evaluated by electrophoresis on a 1% agarose gel. The 16S V4 region was amplified by PCR using the primer 515F/806R of the 16S rRNA gene. Then, the purified PCR products were formed into a library with Ion Plus Fragment Library Kit 48 rxns (Thermo Fisher Scientific, United States) and sequenced on the IonS5 XL platform (Thermo Fisher Scientific, United States) using single-end sequencing. The primers contained adapter sequences and single-end barcodes, which allowed pooling and direct sequencing of PCR products. Cutadapt (V1.9.1)^1^ was applied to the high-quality 16S rRNA gene reads. Subsequently, the 16S rRNA gene read pairs were demultiplexed based on their unique barcodes, and the reads were merged using VSEARCH (Rognes et al., 2016). Sequences were clustered into operational taxonomic units (OTUs) with a similarity cutoff value of 97%. OTU representative sequences were produced using Uparse v7.0.1001. Species annotation analysis was performed on the OTU representative sequences through the SILVA database (SILVA database).^2^ These samples were used for downstream analyses of alpha-diversity, beta-diversity, species composition, between-group variance, and correlation analysis in R v4.0.2.

### Statistical Analysis

The 16S rRNA sequencing data were analyzed using the Kruskal-Wallis test, followed by the Wilcoxon rank-sum test to assess significant differences between the different treatments.

Normality was evaluated using the Shapiro-Wilk normality test. If the data were not normally distributed, they were log-transformed for analysis. Data that remained non-normally distributed were analyzed using the Kruskal-Wallis test followed by the Wilcoxon rank-sum test. Normally distributed data were analyzed using one-way ANOVA followed by the least significant difference (LSD) test. Differences were considered statistically significant at *P* < 0.05. Differences were counted using IBM SPSS Statistics 25.0 and R v4.0.2.

## Results

### The Diversity and Composition of Gut Microbial Community

**FIGURE 2:**
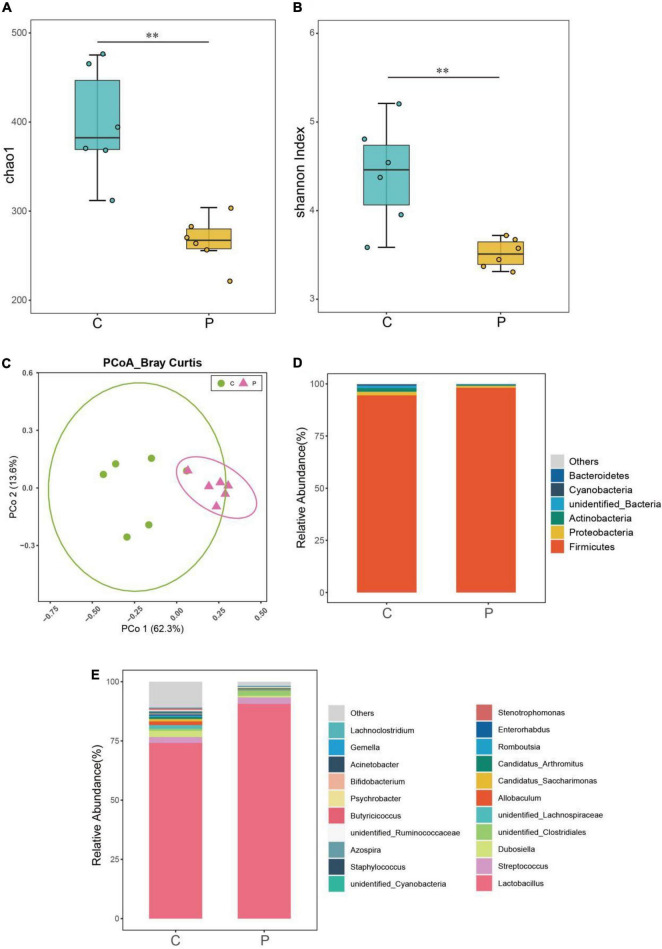
Effects of *L. johnsonii* BS15 on the gut microbial diversity and composition in BS15-pretreated mice. Microbial **(A)** richness (Chao1) and **(B)** evenness (Shannon indexes) in ileal samples of each group. The difference in alpha diversity index was analyzed by Kruskal-Wallis test followed by Wilcoxon rank-sum test. **P < 0.01 represents extremely significant differences between groups. **(C)** Principal coordinate analysis (PCoA) of Bray-Curtis distances between groups with or without BS15. The taxa of high relative abundance (>1%) at the **(D)** phylum level and **(E)** genus level of each group.

Through 16s rRNA gene sequencing, we determined whether *L. johnsonii* BS15 supplementation influenced the gut microbiota. As shown in Figures 2A,B, the Shannon and Chao1 indices of the ileal microbiome in the P group were significantly lower than those of the control group (*P* < 0.01). Moreover, the high relative abundances of the other genera in the P group were different from those in the control group. Moreover, principal coordinate analysis (PCoA) based on Bray-Curtis showed a clear separation (Figure 2C) between the ileal microbiota of the control and P groups. Additionally, the ileal microbial communities in these two groups were dominated by Firmicutes at the phylum level (Figure 2D). The relative abundance of Firmicutes in the P group was significantly higher than that in the control group (Figure 2E). Conversely, the relative abundance of Actinobacteria, Bacteroidetes, and Proteobacteria decreased markedly in the P group compared with that in the control group. At the genus level (Figure 2D), *Lactobacillus* was the most abundant species in the control and P groups.

**FIGURE 3:**
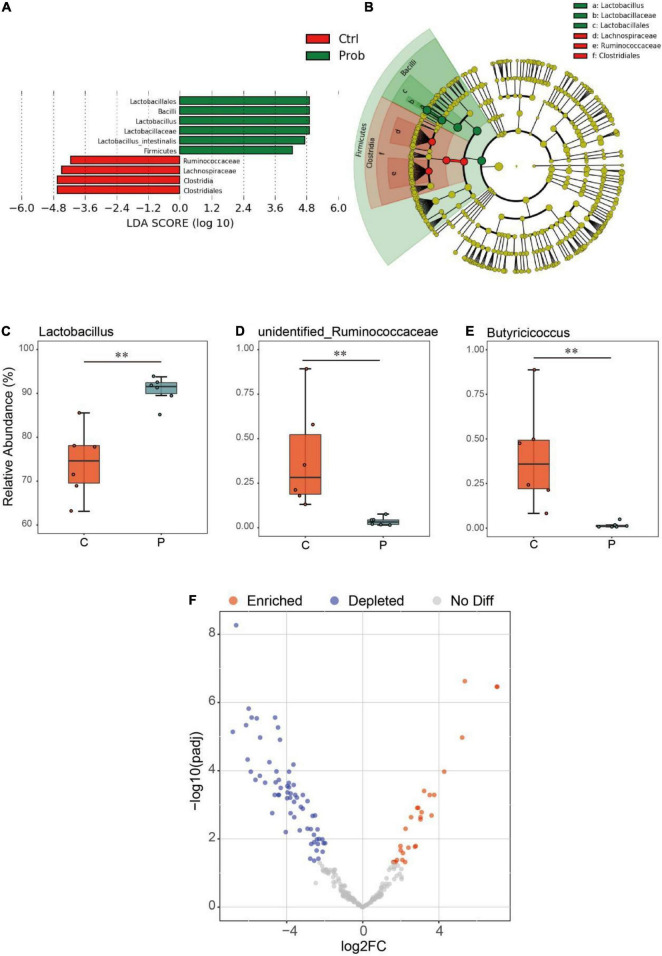
Differential analysis of ileal microbiota in mice. The **(A)** linear discriminant analysis (LDA) score and **(B)** cladogram were generated from LDA effect size (LEfSe). Relative abundance (%) of the **(C)** *Lactobacillus*, **(D)** unidentified Ruminococcaceae, and **(E)** *Butyricicoccus* in each group. **P < 0.01 represents extremely significant differences between groups.**(F)** Enrichment and depletion of the significant differentiated OTUs included in the ileal microbiome. Each point represents an individual species.

As shown in Figures 3A,B, compared with the control and P groups, the biomarkers in the control group were Ruminococcaceae, Lachnospiraceae, *Clostridia*, and Clostridiales, and those in the P group were Lactobacillales, Bacilli, *Lactobacillus*, Lactobacillaceae, *Lactobacillus intestinalis*, and Firmicutes. Compared with the C group, the relative abundance of *Lactobacillus* in the P group was significantly increased (Figure 3C, *P* < 0.01), and the relative abundance of unidentified Ruminococcaceae and *Butyricicoccus* of P group in the ileum decreased significantly (Figures 3D,E, *P* < 0.01). It was possible to quantify the microbial effects of *L. johnsonii* BS15 by analyzing significant differences in OTU abundance relative to the original ileal species (Figure 3F). Under the influence of *L. johnsonii* BS15, partial OTUs were enriched or competitively excluded in the ileum. For instance, 24 OTUs were significantly higher in the P group than that in the control group.

### Effect of BS15 on Ethanol Resistance and Unconsciousness Time Following Acute Ethanol Exposure in Mice

**FIGURE 4:**
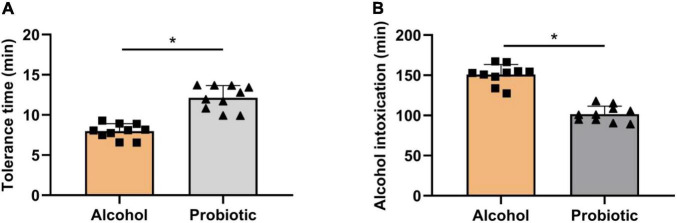
Role of BS15 on resistance and unconsciousness time following acute EtOH exposure in mice. Data are displayed with the mean ± SD (*n* = 10). **(A)** Ethanol resistance time was measured as the interval between acute EtOH exposure and loss of righting reflex. **(B)** Ethanol unconsciousness time was measured as the duration of loss of righting reflex. Significant change between different treatments in same time point is expressed on the basis of *T*-test. Significant difference is shown at *P < 0.05.

The tolerance time (Figure 4A) and unconsciousness time (Figure 4B) were significantly altered by BS15 in the P group. In contrast to the A group, mice pretreated with *L. johnsonii* BS15 showed significantly prolonged body-righting reflex disappearance and shortened unconsciousness time (*P* < 0.05).

### Effect of BS15 on Alcohol Metabolism Following Acute Ethanol Exposure in Mice

**FIGURE 5:**
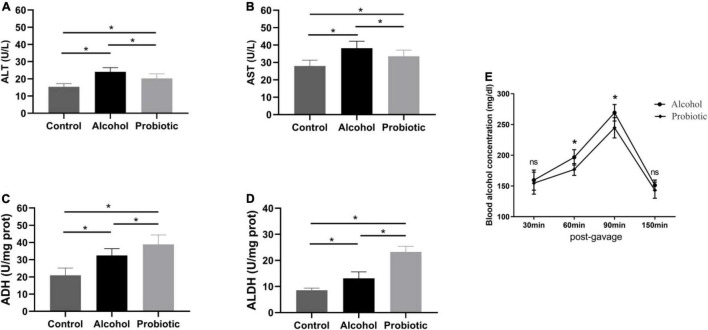
Effect of BS15 on ethanol metabolism following acute EtOH exposure in mice. Data are displayed with the mean ± SD (*n* = 6). **(A)** ALT and **(B)** AST activities in serum following acute EtOH exposure in mice. **(C)** ADH and **(D)** ALDH activities in liver following acute EtOH exposure in mice. Significant difference between groups is expressed on the basis of one-way ANOVA statistical analysis followed by LSD test. **(E)** Blood ethanol concentration following acute EtOH exposure in mice. Significant change between different treatments in same time point is expressed on the basis of *T*-test. Significant difference is shown at *P < 0.05. AST, aspartate amino transferase; ALT, alanine amino transferase; ADH, alcohol dehydrogenase; ALDH, aldehyde dehydrogenase.

As shown in Figures 5A,B, AST and ALT activities in the serum were significantly elevated in group A compared with those in the control group. *L. johnsonii* BS15 pretreatment markedly decreased AST and ALT activities after acute EtOH exposure (*P* < 0.05). This indicated that pretreatment with *L. johnsonii* BS15 could effectively protect the liver from alcohol-induced injury.

Acute EtOH administration in mice induced a significant increase in ADH activity in the liver (*P* < 0.05), and the elevated ADH activity in BS15 pretreated groups was much higher than that in group A (Figure 5C, *P* < 0.05). A similar tendency was observed for ALDH activity between groups A and P (Figure 5D, *P* < 0.05).

The blood alcohol concentration reached a peak at 90 min after alcohol intake, and compared with the A group, the blood alcohol concentration was significantly lower at 60 and 90 min after alcohol administration in the *L. johnsonii* BS15 pretreated group (Figure 5E, *P* < 0.05).

### Behavioral Tests

**FIGURE 6:**
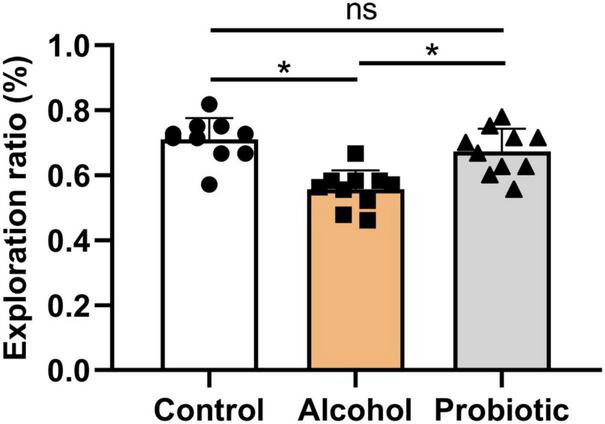
Effects of *L. johnsonii* BS15 on exploration ratio by novel object recognition test. Data are displayed with the mean ± SD (*n* = 10). Significant difference between groups is expressed on the basis of one-way ANOVA statistical analysis followed by LSD test (*P < 0.05). The “ns” means there is no significant difference between groups.

**FIGURE 7:**
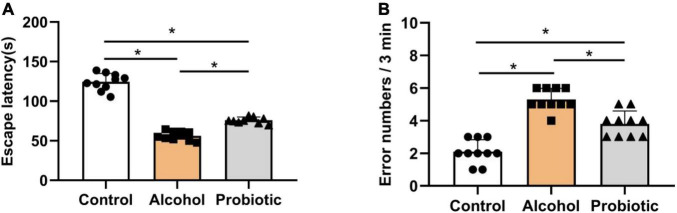
Effects of *L. johnsonii* BS15 on the escape latency and error numbers by passive avoidance test. Data are displayed with the mean ± SD (*n* = 10). **(A)** Escape latency was the interval when the animal walked off the platform for the first time. **(B)** Error number was the total number of times the mice left the platform within 3 min. Significant difference between groups is expressed on the basis of one-way ANOVA statistical analysis followed by LSD test (*P < 0.05).

**FIGURE 8:**
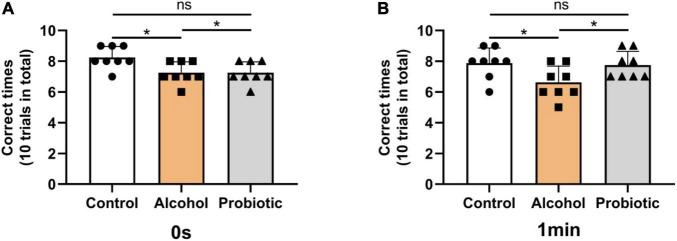
Effects of *L. johnsonii* BS15 on the correct times with both 0 s and 1 min of retention interval by *T*-maze test. Data are displayed with the mean ± SD (*n* = 8). The correct times with **(A)** 0 s or **(B)** 1 min of retention interval by T-maze test. Significant difference between groups is expressed on the basis of one-way ANOVA statistical analysis followed by LSD test (*P < 0.05). The “ns” means there is no significant difference between groups.

Figures 6–8 show the results of the behavioral tests for the memory abilities of the mice. Significantly lower exploration ratio (Figure 6) and escape latency (Figure 7A) were observed (*P* < 0.05) in group A than that in the control group. Correct times for 0 s and 1 min of retention intervals (Figure 8) in group A were also significantly lower (*P* < 0.05) than that in the control group. The error numbers (Figure 7B) in group A were significantly higher than that in the control group. In contrast, the exploration ratio (Figure 6), escape latency (Figure 7A), and correct times for 0 s and 1 min retention intervals (Figure 8) were significantly higher (*P* < 0.05) in the P group. The error numbers (Figure 7B) in group A were significantly lower (*P* < 0.05) than that in group P. Moreover, significant differences (*P* < 0.05) in escape latency and error numbers were observed between the control and P groups (Figure 7), whereas the number of correct responses and exploration ratio were not significant (Figures 6, 8, *P* > 0.05).

### The mRNA Expression of Memory-Related Functional Proteins in the Hippocampus

**FIGURE 9:**
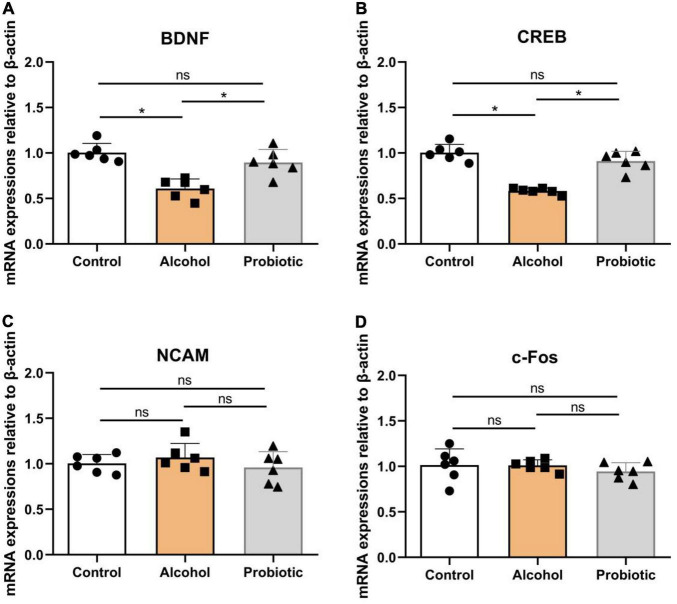
Expression levels of memory-related functional proteins in the hippocampus. **(A–D)** Relative expression of **(A)** BDNF, **(B)** CREB, **(C)** NCAM, and **(D)** c-Fos. Data are displayed with the mean ± SD (*n* = 6). Significant difference between groups is expressed on the basis of one-way ANOVA statistical analysis followed by LSD test (*P < 0.05). The “ns” means there is no significant difference between groups. BDNF, brain-derived neurotrophic factor; CREB, cyclic ampresponse element binding protein; NCAM, neural cell adhesion molecule; c-Fos, immediate early gene.

Figure 9 illustrates the differences in the mRNA expression levels of memory-related functional proteins in the hippocampus among the three groups. The mRNA expression levels of BDNF and CREB were significantly downregulated (Figures 9A,B, *P* < 0.05) in group A compared with those in the control group. However, the mRNA expression levels of NCAM and c-Fos did not change significantly (Figures 9C,D, *P* > 0.05) between these two groups. Moreover, *L. johnsonii* BS15 pretreatment significantly increased (Figures 9A,B, *P* < 0.05) the mRNA expression levels of BDNF and CREB in response to acute alcohol unconsciousness; their mRNA expression levels in the P group were significantly higher (Figures 9A,B, *P* < 0.05) than that in the A group. However, the mRNA expression levels of NCAM and c-Fos remained unchanged (Figures 9C,D, *p* > 0.05) between the control and P groups.

### Antioxidant Capacity in the Hippocampus

**FIGURE 10:**
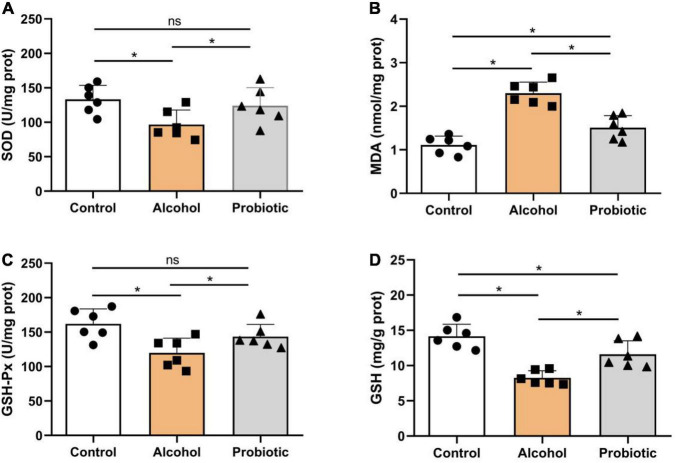
Oxidation and antioxidant levels in the hippocampus. Activities or contents of **(A)** SOD, **(B)** MDA, **(C)** GSH-Px, and **(D)** GSH. Data are displayed with the mean ± SD (*n* = 6). Significant difference between groups is expressed on the basis of one-way ANOVA statistical analysis followed by LSD test (*P < 0.05). The “ns” means there is no significant difference between groups. SOD, superoxide dismutase; MDA, malondialdehyde; GSH-Px, glutathione peroxidase; GSH, glutathione.

Figure 10 illustrates the antioxidant indices in the hippocampus. As shown in Figure 10A, SOD levels were significantly lower (*P* < 0.05) in group A than that in the control group, but no difference (*P* > 0.05) was observed between the control and P groups. Figure 10B shows higher (*P* < 0.05) MDA content in group A than that in the other two groups, while the MDA content in the P group was higher (*P* < 0.05) than that in the control group. Meanwhile, the activity of GSH-Px was significantly lower (*P* < 0.05) in group A (Figure 10C) than that in the control group, but no significant difference (*P* > 0.05) was observed between groups A and P. Moreover, GSH content was significantly decreased (*P* < 0.05) in group A (Figure 10D), but showed no differences (*P* > 0.05) compared with that in the control and P groups.

## Discussion

In earlier studies on the pathology of organs with regard to excessive drinking, most studies focused on changes in physiological and biochemical parameters of the liver, brain, or gut tissue itself (Bajaj, 2019; Carbia et al., 2021). The motivation of this study was to explore the mechanism of memory dysfunction induced by acute EtOH exposure by observing changes in the intestinal flora. Our study also verified the preventative influence of *L. johnsonii* BS15 on drunkenness. The normal animal memory level relies upon a delicate change in the intestinal flora, organizational barrier, and circulation system (Sun et al., 2020; Xin et al., 2020, 2021a; Wang et al., 2021). Although various factors, such as neuroinflammation, lipid peroxidation, autophagy, and apoptosis, can affect brain health, the gut microbiome plays a significant role as one of the main drivers.

16S rRNA high-throughput sequencing was performed on the ileal microbiome to determine whether the structure of the gut microbiota was altered by *L. johnsonii* BS15 supplementation. According to the results shown in the Shannon index and Chao1 index, the bacteria richness was significantly decreased. The above observation indicates that the administration of *L. johnsonii* BS15 stabilizes the intestinal flora. This conclusion is consistent with the results of previous studies, which suggest that *Lactobacillus* can interfere with the normal colonization of other bacteria, especially pernicious bacteria, owing to its enzymatic, fibrinolytic, and broad-spectrum antibacterial activities (Eom et al., 2015). Sepp et al. (2018) found that *Lactobacillus* ME-3 contributed to the species richness, diversity, and abundance of *Lactobacillus* in the intestinal tract, which was related to the increase in the diversity of the entire intestinal flora in 71 volunteers. Moreover, as shown in the PCoA of Bray Curtis distances, we observed an apparent inconsistency between the C and P groups, which manifested the compositional differences of the gut microbiota in probiotic-pretreated mice. Therefore, we believe that the reduction of gut microbiota diversity proves the main effect on intestinal microbiota by BS15 supplementation, and the different microbiota structures could be beneficial to the health of the host.

The ileal microenvironment mainly contributes to the normal colonization of microbes from seven predominant taxa, namely, Firmicutes, Bacteroidetes, Actinobacteria, Fusobacteria, Proteobacteria, Verrucomicrobia, and Cyanobacteria. Among them, Firmicutes, which accounted for up to 90% of the relative abundance, was the dominant phylum in all treatments. Normal intestinal microbes of the same strain of animals have shown different compositional patterns in various studies (Cryan et al., 2019). Even genetically identical rodents may have different microbiota owing to environmental factors, including diet, garbage, suppliers, transportation, and facilities (Knight et al., 2018). Based on our previous findings, mouse models reared under certain experimental conditions showed similar microbial composition characteristics in the gut, such as a high relative abundance of Firmicutes and *Lactobacillus* (Xin et al., 2021a,b). Although these results differ from those of other studies, reconciling microbiome data generated using different methods remains an unsolved challenge. The intestinal microbial community in the BS15 supplement was primarily manifested by an increase in the relative abundance of Firmicutes at the phylum level and *Lactobacillus* at the genus level. *Lactobacillus* is an important genus in Firmicutes and is also a well-known intestinal probiotic. *Lactobacillus* not only resisted colonization by pathogenic bacteria but also was significantly related to the protein expression level of TNF-α, which supported that it is an anti-inflammatory bacterium (Ait-Belgnaoui et al., 2014; Roychowdhury et al., 2018). Therefore, *Lactobacillus* is actively used for the treatment of various intestinal disorders, including alcoholic liver disease (Lovinger, 1997). A growing body of evidence has revealed a frequent rate of psychological and psychiatric problems among patients suffering from alcohol abuse, and these changes in the central nervous system due to the concept of the gut-brain axis are often accompanied by dysbacteriosis. Yan et al. (2011) reported that alcoholic dysbiosis is characterized by reduced proportions of commensal probiotic bacteria such as *Lactobacillus* species in animal models and humans. In our previous studies, *L. johnsonii* BS15, a potential psychobiotic, was shown to have an impact on higher nervous functions, including behavior, which supports the hypothesis that the gut microbiota may play a beneficial role in alcohol-related diseases (Wang et al., 2020, 2021).

Once consumed, alcohol is absorbed mainly in the gastrointestinal tract by diffusion, enters the liver *via* the portal vein, and is distributed rapidly throughout the body. However, as the primary site for alcohol absorption, mounting evidence shows that the GI tract can be affected by alcohol and its metabolites, which experience symptoms such as intestinal bacterial dysbiosis and disruption of tissue homeostasis (Engen et al., 2015). However, probiotics, the key regulators of the intestinal environment, may become a new target for alcoholism. An early study reported an obvious alteration in the intestinal microbiome in rats chronically fed alcohol, but they could benefit from these changes by supplementing rats with *Lactobacillus* GG (Mutlu et al., 2009). Furthermore, alcohol-mediated tissue injury is commonly accompanied by increased intestinal permeability (Tang et al., 2015). Probiotics, such as *Lactobacillus* (Chen et al., 2016) and *Bifidobacterium* (Ewaschuk et al., 2008), or candidate probiotics, such as *Akkermansia* (Everard et al., 2013), have been shown to enhance intestinal integrity through various mechanisms, such as upregulation of tight junction protein expression, improvement of intestinal villus/crypt histology, and thickening of the mucous layer. In this study, *L. johnsonii* BS15 pretreatment significantly prolonged the resistance time and shortened the unconsciousness time in mice after acute EtOH exposure. These results suggest that amelioration of the gut environment by probiotic supplementation may represent a promising and safe approach to promote alcohol metabolism and increase tolerance to alcoholism.

To gain further insight into the degradation mechanism of probiotics, we detected the related enzymes in mice by biochemical analysis. The body typically processes ingested alcohol through a process called oxidative conversion, which predominantly occurs in the liver. Almost 80–90% of EtOH is metabolized by hepatic metabolic enzymes, which are considered necessary for the metabolism of EtOH, including alcohol dehydrogenase (ADH) and acetaldehyde dehydrogenase (ALDH). In short, ADH converts alcohol into acetaldehyde, and ALDH converts acetaldehyde into acetate. Previous studies have suggested that these enzymes can be expressed by some probiotics (Jing et al., 2018). Similarly, Chen et al. (2015) showed that the absence of the intestinal microbiome in germ-free mice was related to the modulation of the gut and hepatic expression of ethanol-metabolizing enzymes. In this study, we showed that the activities of ADH and ALDH were markedly enhanced after acute EtOH exposure, which is considered an adaptive response to alcohol stimulation. Administration of BS15 further upregulated ADH and ALDH activities in liver tissue after EtOH administration. Based on these facts, we speculated that alcohol metabolism could be accelerated by BS15 pretreatment, which is consistent with the obviously suppressed peaking of the blood alcohol concentration. These results indicate that *L. johnsonii* BS15 mediates EtOH metabolism by increasing the activity of related metabolic enzymes. Ferrere et al. (2017) reported that EtOH-fed mice showed a downregulation of the relative abundance of *Bacteroides*, and fecal microbiota transplantation (FMT) from healthy animals markedly reduced EtOH-induced liver injury. In this study, hepatic injury was determined by detecting aspartate aminotransferase (AST) and alanine aminotransferase (ALT) activities in the blood, which are considered the most sensitive indices for hepatic injury. *L. johnsonii* BS15 pretreatment significantly improved AST and ALT activities after acute EtOH intake. These results demonstrate that supplementation with *L. johnsonii* B15 could improve liver impairment under acute EtOH exposure.

In addition to negatively affecting the liver, alcohol impairs brain function, behavior, and cognition. In this study, three behavioral tasks were used to assess hippocampus-associated memory impairments. The T-maze is an elevated or enclosed equipment in the form of a horizontally placed T, which is used in a variety of ways to evaluate the cognitive and memory capability of an animal (Deacon and Rawlins, 2006). NOR is an efficient and flexible assay for investigating various aspects of learning and memory in animals (Lueptow, 2017). The main advantage of NOR is that it relies on rodents’ natural proclivity to explore novelty (Berlyne, 1950). Finally, the passive avoidance test has been widely employed in studies on learning and memory in experimental animals (Jarvik and Essman, 1960). Poor memory ability can be determined by short escape latency and high error numbers (Malekmohamadi et al., 2007; Chen et al., 2014). Notably, the behavioral performance of mice with acute alcohol intake was significantly ameliorated by BS15 pretreatment; thus, the positive effects of *L. johnsonii* BS15 prove it as a potentially beneficial bacterium. The changes in the behavioral tests induced by *L. johnsonii* BS15 pretreatment were consistent with the conclusions of our earlier research (Sun et al., 2020; Xin et al., 2020; Wang et al., 2021).

The hippocampus is an essential brain area for cognitive and memory capabilities and is particularly vulnerable to the damaging effects of acute EtOH exposure (Agartz et al., 1999; Sullivan and Pfefferbaum, 2005; Beresford et al., 2006). Mounting evidence has shown that hippocampal impairment can lead to memory impairment (Guimarães et al., 1993; Molteni et al., 2002). We observed alterations in some pivotal memory-associated functional proteins and antioxidant capacity in hippocampal tissue to elucidate the mechanism underlying the promising performance of *L. johnsonii* BS15. While the mechanisms of alcohol-induced memory deficits are not completely understood, increased oxidative stress may be a major factor contributing to selective neuronal impairment and cognitive deficits secondary to EtOH abuse (Haorah et al., 2008; Crews and Nixon, 2009). Lipid peroxidation is a critical process in molecular injury during oxidative stress that induces hippocampal-dependent memory impairment. The reactive oxygen species generated by stress are responsible for lipid peroxidation, which is determined by the upregulation of MDA formation (Niki, 2012). CAT, SOD, and GSH-Px are important enzymes that protect against oxidative stress by decreasing the activity of superoxide anions and hydrogen peroxide (Thakare et al., 2017). In our study, acute EtOH exposure downregulated the activities or content of SOD, GSH-Px, and GSH and upregulated MDA formation in the hippocampal tissue, implying an improvement in oxidative stress to a certain extent related to alcohol-induced memory impairment. Furthermore, *L. johnsonii* BS15 pretreatment markedly ameliorated EtOH-induced memory disruption, and this effect may be attributed to accelerated EtOH clearance (McGregor, 2007) and attenuation of oxidative stress by increased antioxidant enzyme activities in the hippocampal tissue.

Further studies are needed to identify the exact mechanism by which key intestinal microbes directly or indirectly mediate EtOH-induced memory function damage. Since it is quite difficult to identify key species based only on 16S rDNA high-throughput sequencing, more technology (such as metagenomics) should be considered in future studies to increase the possibility of eventual identification of these species. Meanwhile, we need a specific application of the above treatment method, requiring further studies with greater grouping design, from multiple bowels, across different time points, and accounting for the alteration of metabolic material in the gut, blood, and injured organs.

## Conclusion

These findings indicate that *L. johnsonii* BS15 has beneficial effects against excessive alcohol intake-induced impairment of memory functions and activities of oxidative stress-related enzymes. These alterations could be caused by the improved microbial microenvironment in the gut and alcohol metabolic abilities in the serum and liver of mice. This study deepens our understanding of the link between memory function and the intestinal microenvironment under acute EtOH exposure and provides insights for the therapy of future alcoholism.

## Data Availability Statement

The datasets presented in this study can be found in online repositories. The names of the repository/repositories and accession number(s) can be found below: https://www.ncbi.nlm.nih.gov/search/all/?term=PRJNA791154.

## Ethics Statement

The animal study was reviewed and approved by the Institutional Animal Care and Use Committee of Sichuan Agricultural University (approval number: SYXKchuan2019–187).

## Author Contributions

NS, JX, and HW: conceptualization and methodology. NS, BG, and XC: project administration and data curation. NS and LL: writing—original draft. NS and HW: writing—review and editing. XN, HW, DZ, JF, KP, BJ, YZ, CL, LZ, and PX: supervision. All authors contributed to the article and approved the submitted version.

## Conflict of Interest

JX and HW were employed by the Guangzhou Beneco Biotechnology Co., Ltd. The remaining authors declare that the research was conducted in the absence of any commercial or financial relationships that could be construed as a potential conflict of interest.

## Publisher’s Note

All claims expressed in this article are solely those of the authors and do not necessarily represent those of their affiliated organizations, or those of the publisher, the editors and the reviewers. Any product that may be evaluated in this article, or claim that may be made by its manufacturer, is not guaranteed or endorsed by the publisher.

## Footnotes

1
http://cutadapt.readthedocs.io/en/stable/

2
http://www.arb-silva.de/

## References

[B1] Agartz I. Momenan R. Rawlings R. R. Kerich M. J. Hommer D. W. (1999). Hippocampal volume in patients with alcohol dependence. *Arch. Gen. Psychiatry* 56 356–363. 10.1001/archpsyc.56.4.356 10197833

[B2] Ait-Belgnaoui A. Colom A. Braniste V. Ramalho L. Marrot A. Cartier C. (2014). Probiotic gut effect prevents the chronic psychological stress-induced brain activity abnormality in mice. *Neurogastroenterol. Motil.* 26 510–520. 10.1111/nmo.12295 24372793

[B3] Alasmari F. Goodwani S. McCullumsmith R. E. Sari Y. (2018). Role of glutamatergic system and mesocorticolimbic circuits in alcohol dependence. *Prog. Neurobiol.* 171 32–49. 10.1016/j.pneurobio.2018.10.001 30316901 PMC6261463

[B4] Antunes M. Biala G. (2012). The novel object recognition memory: neurobiology, test procedure, and its modifications. *Cogn. Process.* 13 93–110. 10.1007/s10339-011-0430-z 22160349 PMC3332351

[B5] Asrani S. K. Devarbhavi H. Eaton J. Kamath P. S. (2019). Burden of liver diseases in the world. *J. Hepatol.* 70 151–171. 10.1016/j.jhep.2018.09.014 30266282

[B6] Bajaj J. S. (2019). Alcohol, liver disease and the gut microbiota. *Nat. Rev. Gastroenterol. Hepatol.* 16 235–246. 10.1038/s41575-018-0099-1 30643227

[B7] Beresford T. P. Arciniegas D. B. Alfers J. Clapp L. Martin B. Du Y. (2006). Hippocampus volume loss due to chronic heavy drinking. *Alcohol. Clin. Exp. Res.* 30 1866–1870. 10.1111/j.1530-0277.2006.00223.x 17067350

[B8] Berlyne D. E. (1950). Novelty and curiosity as determinants of exploratory behavior. *Br. J. Psychol.* 41 68–80. 10.1111/j.2044-8295.1950.tb00262.x

[B9] Bravo J. A. Forsythe P. Chew M. V. Escaravage E. Savignac H. M. Dinan T. G. (2011). Ingestion of *Lactobacillus* strain regulates emotional behavior and central GABA receptor expression in a mouse via the vagus nerve. *Proc. Natl. Acad. Sci. U.S.A.* 108 16050–16055. 10.1073/pnas.1102999108 21876150 PMC3179073

[B10] Carbia C. Lannoy S. Maurage P. López-Caneda E. O’Riordan K. J. Dinan T. G. (2021). A biological framework for emotional dysregulation in alcohol misuse: from gut to brain. *Mol. Psychiatry* 26 1098–1118. 10.1038/s41380-020-00970-6 33288871

[B11] Carson E. J. Pruett S. B. (1996). Development and characterization of a binge drinking model in mice for evaluation of the immunological effects of ethanol. *Alcohol. Clin. Exp. Res.* 20 132–138. 10.1111/j.1530-0277.1996.tb01055.x 8651442

[B12] Chen P. Miyamoto Y. Mazagova M. Lee K. C. Eckmann L. Schnabl B. (2015). Microbiota protects mice against acute alcohol-induced liver injury. *Alcohol. Clin. Exp. Res.* 39 2313–2323. 10.1111/acer.12900 26556636 PMC4712135

[B13] Chen R. C. Xu L. M. Du S. J. Huang S. S. Wu H. Dong J. J. (2016). *Lactobacillus rhamnosus* GG supernatant promotes intestinal barrier function, balances Treg and TH17 cells and ameliorates hepatic injury in a mouse model of chronic-binge alcohol feeding. *Toxicol. Lett.* 241 103–110. 10.1016/j.toxlet.2015.11.019 26617183

[B14] Chen X. Cai F. Guo S. Ding F. He Y. Wu J. (2014). Protective effect of Flos puerariae extract following acute alcohol intoxication in mice. *Alcohol. Clin. Exp. Res.* 38 1839–1846. 10.1111/acer.12437 24931816

[B15] Crews F. T. Nixon K. (2009). Mechanisms of neurodegeneration and regeneration in alcoholism. *Alcohol. Alcohol.* 44 115–127. 10.1093/alcalc/agn079 18940959 PMC2948812

[B16] Cryan J. F. O’Riordan K. J. Cowan C. S. M. Sandhu K. V. Bastiaanssen T. F. S. Boehme M. (2019). The microbiota-gut-brain axis. *Physiol. Rev.* 99 1877–2013. 10.1152/physrev.00018.2018 31460832

[B17] Deacon R. M. J. Rawlins J. N. P. (2006). T-maze test alternation in the rodent. *Nat. Protoc.* 1:712. 10.1038/nprot.2006.2 17406205

[B18] Engen P. A. Green S. J. Voigt R. M. Forsyth C. B. Keshavarzian A. (2015). The gastrointestinal microbiome: alcohol effects on the composition of intestinal microbiota. *Alcohol. Res.* 37 223–236. 26695747 10.35946/arcr.v37.2.07 PMC4590619

[B19] Eom J. S. Song J. Choi H. S. (2015). Protective effects of a novel probiotic strain of *Lactobacillus plantarum* JSA22 from traditional fermented soybean food against infection by *Salmonella enterica Serovar typhimurium*. *J. Microbiol. Biotechnol.* 25 479–491. 10.4014/jmb.1501.01006 25639720

[B20] Everard A. Belzer C. Geurts L. Ouwerkerk J. P. Druart C. Bindels L. B. (2013). Cross-talk between *Akkermansia muciniphila* and intestinal epithelium controls diet-induced obesity. *Proc. Natl. Acad. Sci. U.S.A.* 110 9066–9071. 10.1073/pnas.1219451110 23671105 PMC3670398

[B21] Ewaschuk J. B. Diaz H. Meddings L. Diederichs B. Dmytrash A. Backer J. (2008). Secreted bioactive factors from *Bifidobacterium infantis* enhance epithelial cell barrier function. *Am. J. Physiol. Gastrointest. Liver Physiol.* 295 G1025–G1034. 10.1152/ajpgi.90227.2008 18787064

[B22] Fang P. Kazmi S. A. Jameson K. G. Hsiao E. Y. (2020). The microbiome as a modifier of neurodegenerative disease risk. *Cell Host Microbe* 28 201–222. 10.1016/j.chom.2020.06.008 32791113 PMC7430034

[B23] Ferrere G. Wrzosek L. Cailleux F. Turpin W. Puchois V. Spatz M. (2017). Fecal microbiota manipulation prevents dysbiosis and alcohol-induced liver injury in mice. *J. Hepatol.* 66 806–815. 10.1016/j.jhep.2016.11.008 27890791

[B24] Gareau M. G. Wine E. Rodrigues D. M. Cho J. H. Whary M. T. Philpott D. J. (2011). Bacterial infection causes stress-induced memory dysfunction in mice. *Gut* 60 307–317. 10.1136/gut.2009.202515 20966022

[B25] Gong Y. S. Hou F. L. Guo J. Lin L. Zhu F. Y. (2021). Effects of alcohol intake on cognitive function and β-amyloid protein in APP/PS1 transgenic mice. *Food Chem. Toxicol.* 151:112105. 10.1016/j.fct.2021.112105 33737111

[B26] Guimarães F. S. Del Bel E. A. Padovan C. M. Netto S. M. de Almeida R. T. (1993). Hippocampal 5-HT receptors and consolidation of stressful memories. *Behav. Brain Res.* 58 133–139. 10.1016/0166-4328(93)90098-b 8136041

[B27] Hao Z. Wang W. Guo R. Liu H. (2019). *Faecalibacterium prausnitzii* (ATCC 27766) has preventive and therapeutic effects on chronic unpredictable mild stress-induced depression-like and anxiety-like behavior in rats. *Psychoneuroendocrinology* 104 132–142. 10.1016/j.psyneuen.2019.02.025 30844607

[B28] Haorah J. Ramirez S. H. Floreani N. Gorantla S. Morsey B. Persidsky Y. (2008). Mechanism of alcohol-induced oxidative stress and neuronal injury. *Free Radic Biol. Med.* 45 1542–1550. 10.1016/j.freeradbiomed.2008.08.030 18845238 PMC2605399

[B29] Hillemacher T. Bachmann O. Kahl K. G. Frieling H. (2018). Alcohol, microbiome, and their effect on psychiatric disorders. *Prog. Neuropsychopharmacol. Biol. Psychiatry* 85 105–115. 10.1016/j.pnpbp.2018.04.015 29705711

[B30] Jarvik M. Essman W. (1960). A simple one-trial learning situation for mice. *Psychol. Rep.* 6:290. 10.2466/PR0.6.2.290-290

[B31] Jing L. Yunbin L. Li M. Jing S. Huang Z. Lu F. (2018). Alleviating acute alcoholic liver injury in mice with *Bacillus subtilis* co-expressing alcohol dehydrogenase and acetaldehyde dehydrogenase. *J. Funct. Foods* 49 342–350. 10.1016/j.jff.2018.09.006

[B32] Knight R. Vrbanac A. Taylor B. C. Aksenov A. Callewaert C. Debelius J. (2018). Best practices for analysing microbiomes. *Nat. Rev. Microbiol.* 16 410–422. 10.1038/s41579-018-0029-9 29795328

[B33] Koob G. F. (2013). Negative reinforcement in drug addiction: the darkness within. *Curr. Opin. Neurobiol.* 23 559–563. 10.1016/j.conb.2013.03.011 23628232

[B34] Liang S. Wang T. Hu X. Luo J. Li W. Wu X. (2015). Administration of *Lactobacillus helveticus* NS8 improves behavioral, cognitive, and biochemical aberrations caused by chronic restraint stress. *Neuroscience* 310 561–577. 10.1016/j.neuroscience.2015.09.033 26408987

[B35] Lovinger D. M. (1997). Serotonin’s role in alcohol’s effects on the brain. *Alcohol. Health Res. World* 21 114–120. 15704346 PMC6826824

[B36] Lowe P. P. Gyongyosi B. Satishchandran A. Iracheta-Vellve A. Cho Y. Ambade A. (2018). Reduced gut microbiome protects from alcohol-induced neuroinflammation and alters intestinal and brain inflammasome expression. *J. Neuroinflammation* 15:298. 10.1186/s12974-018-1328-9 30368255 PMC6203993

[B37] Lueptow L. M. (2017). Novel object recognition test for the investigation of learning and memory in mice. *J. Vis. Exp.* 126:55718. 10.3791/55718 28892027 PMC5614391

[B38] Malekmohamadi N. Heidari P. Sahebgharani M. Zarrindast M. R. (2007). Effects of clozapine and sulpiride on morphine state-dependent memory in the step-down passive avoidance test. *Pharmacology* 79 149–153. 10.1159/000098151 17191034

[B39] McGregor N. R. (2007). *Pueraria lobata* (Kudzu root) hangover remedies and acetaldehyde-associated neoplasm risk. *Alcohol* 41 469–478. 10.1016/j.alcohol.2007.07.009 17980785

[B40] Messaoudi M. Violle N. Bisson J. F. Desor D. Javelot H. Rougeot C. (2011). Beneficial psychological effects of a probiotic formulation (*Lactobacillus helveticus* R0052 and *Bifidobacterium longum* R0175) in healthy human volunteers. *Gut Microbes* 2 256–261. 10.4161/gmic.2.4.16108 21983070

[B41] Mews P. Egervari G. Nativio R. Sidoli S. Donahue G. Lombroso S. I. (2019). Alcohol metabolism contributes to brain histone acetylation. *Nature* 574 717–721. 10.1038/s41586-019-1700-7 31645761 PMC6907081

[B42] Mohammadi A. A. Jazayeri S. Khosravi-Darani K. Solati Z. Mohammadpour N. Asemi Z. (2016). The effects of probiotics on mental health and hypothalamic-pituitary-adrenal axis: a randomized, double-blind, placebo-controlled trial in petrochemical workers. *Nutr. Neurosci.* 19 387–395. 10.1179/1476830515Y.0000000023 25879690

[B43] Molteni R. Barnard R. J. Ying Z. Roberts C. K. Gómez-Pinilla F. (2002). A high-fat, refined sugar diet reduces hippocampal brain-derived neurotrophic factor, neuronal plasticity, and learning. *Neuroscience* 112 803–814. 10.1016/s0306-4522(02)00123-9 12088740

[B44] Morais L. H. Schreiber H. L. IV Mazmanian S. K. (2021). The gut microbiota-brain axis in behaviour and brain disorders. *Nat. Rev. Microbiol.* 19 241–255. 10.1038/s41579-020-00460-0 33093662

[B45] Mutlu E. Keshavarzian A. Engen P. Forsyth C. B. Sikaroodi M. Gillevet P. (2009). Intestinal dysbiosis: a possible mechanism of alcohol-induced endotoxemia and alcoholic steatohepatitis in rats. *Alcohol. Clin. Exp. Res.* 33 1836–1846. 10.1111/j.1530-0277.2009.01022.x 19645728 PMC3684271

[B46] Neyrinck A. M. Etxeberria U. Taminiau B. Daube G. Van Hul M. Everard A. (2017). Rhubarb extract prevents hepatic inflammation induced by acute alcohol intake, an effect related to the modulation of the gut microbiota. *Mol. Nutr. Food Res.* 61:899. 10.1002/mnfr.201500899 26990039

[B47] Niki E. (2012). Do antioxidants impair signaling by reactive oxygen species and lipid oxidation products? *FEBS Lett.* 586 3767–3770. 10.1016/j.febslet.2012.09.025 23022561

[B48] Niu R. Chen H. Manthari R. K. Sun Z. Wang J. Zhang J. (2018). Effects of fluoride on synapse morphology and myelin damage in mouse hippocampus. *Chemosphere* 194 628–633. 10.1016/j.chemosphere.2017.12.027 29241138

[B49] O’Hagan C. Li J. V. Marchesi J. R. Plummer S. Garaiova I. Good M. A. (2017). Long-term multi-species *Lactobacillus* and *Bifidobacterium* dietary supplement enhances memory and changes regional brain metabolites in middle-aged rats. *Neurobiol. Learn. Mem.* 144 36–47. 10.1016/j.nlm.2017.05.015 28602659

[B50] Rognes T. Flouri T. Nichols B. Quince C. Mahé F. (2016). VSEARCH: a versatile open source tool for metagenomics. *PeerJ* 4:e2584. 10.7717/peerj.2584 27781170 PMC5075697

[B51] Roychowdhury S. Cadnum J. Glueck B. Obrenovich M. Donskey C. Cresci G. A. M. (2018). *Faecalibacterium prausnitzii* and a prebiotic protect intestinal health in a mouse model of antibiotic and *Clostridium difficile* exposure. *JPEN J. Parenter. Enteral. Nutr.* 42 1156–1167. 10.1002/jpen.1053 29385239 PMC6068000

[B52] Scholey A. Benson S. Kaufman J. Terpstra C. Ayre E. Verster J. C. (2019). Effects of alcohol hangover on cognitive performance: findings from a field/internet mixed methodology study. *J. Clin. Med.* 8:440. 10.3390/jcm8040440 30935081 PMC6518120

[B53] Sepp E. Smidt I. Tepetova J. Rp T. Hütt P. Rtsep M. (2018). The effect of *Lactobacillus fermentum* ME-3 on the intestinal microbiota and urine polyamines content: a double-blind placebo-controlled pilot trial. *J. Funct. Foods* 48 430–438. 10.1016/j.jff.2018.04.053

[B54] Sharon G. Sampson T. R. Geschwind D. H. Mazmanian S. K. (2016). The central nervous system and the gut microbiome. *Cell* 167 915–932. 10.1016/j.cell.2016.10.027 27814521 PMC5127403

[B55] Smith C. J. Emge J. R. Berzins K. Lung L. Khamishon R. Shah P. (2014). Probiotics normalize the gut-brain-microbiota axis in immunodeficient mice. *Am. J. Physiol. Gastrointest. Liver Physiol.* 307 G793–G802. 10.1152/ajpgi.00238.2014 25190473 PMC4200314

[B56] Stärkel P. Schnabl B. (2016). Bidirectional communication between liver and gut during alcoholic liver disease. *Semin. Liver Dis.* 36 331–339. 10.1055/s-0036-1593882 27997973

[B57] Sullivan E. V. Pfefferbaum A. (2005). Neurocircuitry in alcoholism: a substrate of disruption and repair. *Psychopharmacology* 180 583–594. 10.1007/s00213-005-2267-6 15834536

[B58] Sun N. Ni X. Wang H. Xin J. Zhao Y. Pan K. (2020). Probiotic *Lactobacillus johnsonii* BS15 prevents memory dysfunction induced by chronic high-fluorine intake through modulating intestinal environment and improving gut development. *Probiotics Antimicrob. Proteins* 12 1420–1438. 10.1007/s12602-020-09644-9 32166711

[B59] Tang Y. Zhang L. Forsyth C. B. Shaikh M. Song S. Keshavarzian A. (2015). The role of miR-212 and iNOS in alcohol-Induced intestinal barrier dysfunction and steatohepatitis. *Alcohol. Clin. Exp. Res.* 39 1632–1641. 10.1111/acer.12813 26207424 PMC4558329

[B60] Thakare V. N. Dhakane V. D. Patel B. M. (2017). Attenuation of acute restraint stress-induced depressive like behavior and hippocampal alterations with protocatechuic acid treatment in mice. *Metab. Brain Dis.* 32 401–413. 10.1007/s11011-016-9922-y 27785705

[B61] Vuong H. E. Yano J. M. Fung T. C. Hsiao E. Y. (2017). The microbiome and host behavior. *Annu. Rev. Neurosci.* 40 21–49. 10.1146/annurev-neuro-072116-031347 28301775 PMC6661159

[B62] Wang H. S. Xin J. Zhang T. Sun N. Li L. Ni X. (2021). Psychoactive effects of *Lactobacillus johnsonii* against restraint stress-Induced memory dysfunction in mice through modulating intestinal inflammation and permeability-a study based on the gut-brain axis hypothesis. *Front. Pharmacol.* 12:662148. 10.3389/fphar.2021.662148 34122081 PMC8189558

[B63] Wang H. Sun Y. Xin J. Zhang T. Sun N. Ni X. (2020). *Lactobacillus johnsonii* BS15 prevents psychological stress-induced memory dysfunction in mice by modulating the gut-brain axis. *Front. Microbiol.* 11:1941. 10.3389/fmicb.2020.01941 32903531 PMC7438410

[B64] Warburton E. C. Brown M. W. (2015). Neural circuitry for rat recognition memory. *Behav. Brain Res.* 285 131–139. 10.1016/j.bbr.2014.09.050 25315129 PMC4383363

[B65] World Health Organization (2014). Global status report on alcohol and health 2014. *J. Glob. Health* 18 1–57. 10.1111/tmi.12618 26448195

[B66] Xin J. Wang H. Sun N. Bughio S. Zeng D. Li L. (2021a). Probiotic alleviate fluoride-induced memory impairment by reconstructing gut microbiota in mice. *Ecotoxicol. Environ. Saf.* 215:112108. 10.1016/j.ecoenv.2021.112108 33799132

[B67] Xin J. Sun N. Wang H. Ma H. Wu B. Li L. (2021b). Preventive effects of *Lactobacillus johnsonii* on the renal injury of mice induced by high fluoride exposure: insights from colonic microbiota and co-occurrence network analysis. *Ecotoxicol. Environ. Saf.* 228:113006. 10.1016/j.ecoenv.2021.113006 34826728

[B68] Xin J. Zeng D. Wang H. Ni X. Yi D. Pan K. (2014). Preventing non-alcoholic fatty liver disease through *Lactobacillus johnsonii* BS15 by attenuating inflammation and mitochondrial injury and improving gut environment in obese mice. *Appl. Microbiol. Biotechnol.* 98 6817–6829. 10.1007/s00253-014-5752-1 24811405

[B69] Xin J. Zeng D. Wang H. Sun N. Khalique A. Zhao Y. (2020). *Lactobacillus johnsonii* BS15 improves intestinal environment against fluoride-induced memory impairment in mice-a study based on the gut-brain axis hypothesis. *PeerJ* 8:e10125. 10.7717/peerj.10125 33083147 PMC7547597

[B70] Yan A. W. Fouts D. E. Brandl J. Stärkel P. Torralba M. Schott E. (2011). Enteric dysbiosis associated with a mouse model of alcoholic liver disease. *Hepatology* 53 96–105. 10.1002/hep.24018 21254165 PMC3059122

